# Utilizing Modified Coopland’s Scoring System to Identify and Predict the Outcome of High-Risk Pregnancies in Resource-Limited Settings: A Retrospective Review

**DOI:** 10.7759/cureus.65965

**Published:** 2024-08-01

**Authors:** Bhoomika Biradar, Manoj Mathew, Naveen Ramesh

**Affiliations:** 1 Rural Service, St. John’s Medical College, Bangalore, IND; 2 Community Health, St. John's Medical College, Bangalore, IND

**Keywords:** antenatal screening, low birth weight, preterm birth, perinatal mortality, maternal mortality, modified coopland’s scoring system, high-risk pregnancies

## Abstract

Introduction

A high-risk pregnancy is associated with adverse maternal and foetal outcomes. Women with high-risk pregnancies are at a greater risk of developing antepartum haemorrhage, miscarriages, and the need for surgical interventions. Neonatal complications include preterm births, low birth weight (LBW), intra-uterine deaths and an increased need for NICU admission. The utilisation of low-cost scoring tools for identifying high-risk women can aid in early diagnosis and timely implementation of therapeutic interventions.

Objective

The retrospective record-based study sought to calculate the proportion of high-risk pregnancies using modified Coopland’s scoring system and compare the maternal and foetal outcomes among high-risk pregnancies.

Methods

The study retrospectively analysed the records of antenatal women in their third trimester from the years December 2018 to December 2021. Each record was then numerically assessed according to the modified Coopland's scoring system and categorised according to the risk status. Maternal and neonatal outcomes were then compared across the risk groups.

Results

The data included 300 cases over a three-year period. According to modified Coopland's scoring system, we found that the overall proportion of high-risk pregnancies was 18.3%. Adverse maternal and fetal outcomes were increased in high-risk pregnancy groups when compared to low-risk pregnancies, miscarriages (31.6% vs 15.8%) and antepartum haemorrhage (55.6% vs 11.1%). Babies born to high-risk mothers had a higher chance of developing LBW status (52.0%) and respiratory distress (45.5%) when compared to those born to low-risk mothers: 8.0% and 13.6%, respectively.

Conclusion

A notable portion of pregnant women were classified as high-risk using modified Coopland's scoring tool and would benefit from targeted obstetric care.

## Introduction

Maternal mortality is a matter of grave concern worldwide and even more so in developing nations such as India. According to the World Health Organisation (WHO) report in the year 2020, 810 women die every day due to complications arising from pregnancy and childbirth [1]. Maternal health indicators such as maternal mortality and morbidity are particularly useful in reflecting the quality of health services provided by a country [2].

Nearly 15% of pregnant women worldwide are at risk of developing life-threatening complications contributing to maternal and newborn deaths [3]. In India, this proportion is even higher; nearly 20-30% of pregnancies are high risk, accounting for 75% of perinatal morbidity and mortality [4]. In the year 2000, the maternal mortality rate in India was 370 per 100,00 live births against a global MMR of 342 deaths per 100,000 live births [1]. Studies have shown that the proportion of maternal deaths is higher in lower-income countries as compared to higher-income countries, with nearly 90% of all maternal deaths occurring in the former. In 2017, the MMR in low-income countries was 462 per 100,000 live births as compared to 11 per 100,000 live births in high-income countries [5]. Currently, India has achieved its target set by the National Health Policy for MMR i.e., 97 per 100,000 live births in 2018-2020. However, despite this decline, India contributed to 12% of maternal deaths globally in 2017 [1,6]. The main contributors to these maternal deaths ranged from complications arising due to blood loss, infections, high blood pressure developed during pregnancy and abortions carried out in unsafe conditions [7]. 

Many of the maternal deaths are preventable by early identification of pregnancies that are at a higher risk of adverse events. A high-risk pregnancy (HRP) is defined as one that threatens the health or life of the mother or her foetus [8]. Women with HRPs are at a greater risk of developing adverse maternal outcomes such as miscarriages, infection, antenatal haemorrhage, need for surgical interventions, and an increased risk of maternal mortality. Neonatal risks include intra-uterine deaths (IUDs), preterm births, low birth weight (LBW) status, respiratory distress and an increased need for admission to the NICU [9-15].

It is imperative to administer targeted antenatal services by utilisation of methods capable of identifying HRPs for early intervention [16]. One such method that is helpful in the diagnosis of HRPs is the utilisation of screening tools such as scoring systems which assign weighted values according to various risk factors based on maternal and past obstetric history, as well as any complications developed during the present pregnancy [9-10,17]. The assigned scores are then used for risk stratification. This is especially significant in healthcare centres with limited resources in India as it is easy to access, convenient to administer and cost-effective.

## Materials and methods

The study was conducted in the Department of Obstetrics and Gynaecology at a secondary level health care private facility located in Krishnagiri, Tamil Nadu, India. This is a retrospective case-record-based study which included 300 women who availed antenatal care from December 2018 and December 2021. Institutional Ethics approval was obtained along with permission from the hospital before initiation of data collection (IEC 30/2022).

The inclusion criteria were pregnant women (16 to 45 years of age) who had a minimum of six antenatal check-ups at the above-mentioned healthcare facility. The records of all antenatal women were studied and details regarding their medical and obstetric history were noted. We utilised modified Coopland’s scoring system as a scoring tool to numerically assess and assign a score to each record, thereby categorising records according to the risk status.

The scoring system consists of three parts. Part A included risk factors based on age and obstetric status, Part B assigned scores according to pre-existing medical or surgical conditions such as chronic hypertension, heart disease, and previous gynaecological surgery, and Part C described current complications developed during the present pregnancy, including history of bleeding, malpresentation, pre-eclampsia (PE) and gestational diabetes (GDM). Subsequently, the records were grouped into three main categories according to their scores which were assessed in the third trimester - low risk (0-3), moderate risk (4-6) and high risk (>7) [17].

The study aimed to calculate the proportion of HRPs among antenatal women presenting to the hospital in the past three years and to compare the maternal and foetal outcomes among HRPs. The maternal outcomes included the outcome of pregnancy, complications developed during pregnancy, mode of delivery, postpartum haemorrhage (PPH), and maternal mortality. The main foetal outcomes included preterm birth (<37 weeks gestation), LBW (<2.5kgs at birth), IUDs, need for NICU admission, meconium aspiration syndrome (MAS) and congenital anomalies. Additionally, socio-demographic factors such as age and educational status, and obstetric factors (parity) were analysed.

The data was entered in Microsoft Excel and analysed using IBM SPSS Statistics for Windows, Version 22 (Released 2013; IBM Corp., Armonk, New York, United States). The outcome variables were described as frequencies and proportions. The chi-square test of significance or Fisher’s exact test was used to check for association between each of the outcome variables and various risk factors and a p-value of <0.05 was considered statistically significant.

## Results

Three hundred pregnant women had registered during the study time period and all the 300 medical records were included in this study. The distribution of pregnancies following risk stratification was grouped accordingly: high-risk 55(18.3%), moderate-risk 100 (33.3%) and low-risk pregnancies 145(48.3%). The scores ranged from 1 to 16 (3±3.07). 

Socio-demographic factors

The women in our study were in the reproductive age group (16 to 45 years of age). The majority of women belonged to the age group 18 to 35. The mean age was 24.4±3.7 years. In the HRP group, 49 out of 55 (89.0%) women were nulliparous, and this was of statistical significance (p<0.05) (Table 1). 

**Table 1:** Distribution of study population according to the socio-demographic profile Results with a p-value less than 0.05 are considered statistically significant *Chi square test; ^#^Fisher's exact test

Variable		Low risk (n=145) n (%)	Moderate risk (n=100) n (%)	High-risk(n=55) n (%)	Total (n=300) n (%)	p-value
Age (years)	<18	0(0.0%)	1 (100.0%)	0(0.0%)	1 (0.3%)	0.44#
	18-35	145 (48.7%)	98 (32.9%)	55 (18.5%)	298 (99.3%)	
	>35	0 (0.0%)	1 (100.0%)	0 (0.0%)	1 (0.3%)	
Educational status	Primary	1 (50.0%)	0 (0.0%)	1 (50.0%)	2 (0.66%)	0.55#
	Secondary	66 (48.2%)	43 (31.4%)	28 (20.4%)	137(45.6%)	
	Degree	78 (48.4%)	57 (35.4%)	26 (16.1%)	161 (53.6%)	
Parity	0	77 (37.4%)	80 (38.8%)	49 (23.8%)	206 (68.7%)	<0.05*
	1-4	68 (72.3%)	20 (21.3%)	6 (6.4%)	94 (31.3%)	
	>5	0 (0.0%)	0 (0.0%)	0(0.0%)	0 (0.0%)	

Maternal outcomes 

The maternal outcomes are depicted in Table 2. Moderate- and high-risk mothers had an increased risk of undergoing miscarriage (10, 52.6%) (6, 31.6%) respectively as compared to low-risk mothers (3, 15.8%) (p<0.05). Similarly, the risk of bleeding complications such as antepartum haemorrhage 5(55.6%) was observed more in high-risk mothers (p<0.05). High- and moderate-risk pregnancies were associated with an increased need for caesarean sections as a mode of delivery, with 50 out of 55(90%) HRPs requiring caesarean section. There was no maternal mortality during the review period.

**Table 2:** Association between at-risk pregnancy groups and maternal outcomes Results with a p-value less than 0.05 are considered statistically significant *Chi-square test; ^#^Fisher's exact test; NA: Not applicable

Variable		Low-risk (n=145) n (%)	Moderate-risk (n=100) n (%)	High-risk (n=55) n (%)	Total (n=300) n (%)	p-value
Miscarriage	Yes	3 (15.8%)	10 (52.6%)	6 (31.6%)	19 (6.3%)	<0.05#
	No	142 (50.5%)	90 (32.0%)	49 (17.4%)	281 (93.7%)	
Bleeding	Yes	1 (11.1%)	3 (33.3%)	5 (55.6%)	9 (3.0%)	<0.05#
	No	144 (49.5%)	97 (3.3%)	50 (17.2%)	291 (97.0%)	
Caesarian section	Yes	55(31.8%)	74 (42.8%)	44(25.4%)	173 (57.7%)	<0.05*
	No	90 (70.9%)	26 (20.4%)	11 (8.7%)	127 (42.3%)	
PPH	Yes	1 (11.1%)	2 (22.2%)	6(66.7%)	9 (3.0%)	0.66#
	No	144 (49.5%)	98 (33.7%)	49 (16.8%)	291 (97.0%)	
Maternal Mortality		0 (0.0%)	0 (0.0%)	0 (0.0%)		NA

Neonatal outcomes 

LBW status was most predominant in babies born to high-risk mothers 13 (52.0%), followed by moderate-risk mothers 10 (40.0%) when compared to low-risk mothers 2 (<10.0%) (p<0.05). The findings also suggest that HRPs were more likely to result in respiratory distress in babies 10 (45.5%) when compared to low-risk pregnancies 3(13.6%) (p<0.05). Among the 300 pregnancies, 6 resulted in IUDs -3(50.0%) from the high-risk group, 2 (33.3%) from moderate-risk, and 1 (16.7%) from the low-risk groups. As shown in Table 3, two (50.0%) of the newborns were admitted to NICU admission and preterm births 2 (40.0%) were two times higher in high-risk groups when compared to the low-risk group. However, this was not statistically significant (Table 3).

**Table 3:** Association between at-risk pregnancy groups and neonatal outcomes Results with a p-value less than 0.05 are considered statistically significant *Chi square test; ^#^Fisher's exact test

Variable		Low risk (n=145) n (%)	Moderate risk (n=100) n (%)	High-risk (n=55) n (%)	Total (n=300) n (%)	p-value*[DR1] [BB2]
Low birth weight (<2.5kgs)	Yes	2 (8.0%)	10 (40.0%)	13 (52.0%)	25 (8.3%)	<0.05#
	No	143 (42.5%)	90 (32.7%)	42 (15.3%)	275 (91.7%)	
Respiratory distress	Yes	3 (13.6%)	9 (40.9%)	10(45.5%)	22 (7.3%)	<0.05#
	No	142 (51.1%)	91(32.7%)	45 (16.2%)	278 (92.7%)	
Intra-uterine death (IUD)	Yes	1 (16.7%)	2 (33.3%)	3 (50.0%)	6 (2.0%)	0.08#
	No	144 (49.0%)	98 (33.3%)	52 (17.7%)	294 (98.0%)	
NICU admission	Yes	1 (25.0%)	1 (25.0%)	2 (50.0%)	6 (2.0%)	0.25#
	No	144 (49.0%)	99 (33.7%)	53 (18.3%)	294 (98.0%)	
Preterm birth	Yes	1 (20.0%)	2 (40.0%)	2 (40.0%)	5 (1.7%)	0.27#
	No	144 (48.8%)	98 (33.2%)	53 (18.0%)	295 (98.3%)	

## Discussion

The average estimated number of maternal deaths annually in India stands at approximately 24,000 [18]. The Government of India has prioritised maternal and child healthcare and is currently aiming for an MMR of 70 per 100,000 live births by the year 2030, a target set by the United Nations (UN) under the Sustainable Development Goals (SDGs) [19]. To aid in the improvement of health outcomes of pregnant women, the government has adopted the Reproductive, Maternal, New-born, Child and Adolescent Health (RMNCH+A) framework, which includes health programmes such as the Pradhan Mantri Surakshit Matritva Abhiyan (PMSMA) launched in 2016 and Surakshit Matritva Aashwasan (SUMAN) in 2019 [20]. These programmes were developed with the intention of providing high-quality and comprehensive obstetric healthcare to all women in their antenatal and postnatal periods free of cost [20].

Among the significant components of the PMSMA for reducing severe maternal outcomes is the identification of HRPs in order to provide extra attention and intervene when necessary. PMSMA clinics are conducted by an obstetrician and support staff once monthly to screen pregnant mothers for risk factors. Once screened, the women are assigned stickers according to their risk status- green sticker (low-risk) and red sticker (high-risk). In spite of robust screening measures, only less than 15% of total HRPs are reported, contributing to decreased quality of antenatal care [21]. This could be attributed to the fact that the PMSMA clinics rely on screening pregnancies using a limited number of factors in the current pregnancy (anaemia, diabetes, STIs, hypothyroidism, and PE) in order to identify HRPs failing to account for the significant contribution of demographic, past medical and obstetric history in determining high-risk status. In comparison, evidence-based scoring criteria, such as modified Coopland’s system, are a more reliable tool for the identification of high-risk mothers [17]. Thus, in our study, we utilised the modified Coopland’s scoring system to calculate the proportion of HRPs in antenatal women attending the Obstetrics and Gynaecology clinic, and its outcome on the mother as well as the baby.

It was observed that among all the antenatal women attending our clinic, 55 (18.3%) were HRPs. This was similar to a study conducted by Yeoh et al. in which 28% of the pregnancies were high-risk [22]. Higher prevalence was found in studies conducted by Chate et al. (45.9%) [14].

Socio-demographic profiles of the study participants revealed that the majority (99.3%) were between the age group of 18-35 years. In a similar study conducted by Chate et al., 81.6% of the participants belonged to the 20 to 29 age group [14]. There was statistical significance between nulliparity and higher risk pregnancies, out of the total 300 pregnancies, 80 (38.8%) nulliparous women belonged to the moderate-risk category and 49(23.8%) belonged to the high-risk category. In another study conducted across 25 medical centres in the United States, nulliparity was significantly associated with adverse maternal outcomes such as chorioamnionitis, perineal lacerations, and neonatal outcomes such as small for gestational age (SGA) and shoulder dystocia [11]. We found that there was no statistical significance between education status and HRPs and that the majority of our patient population had received an undergraduate level of education. However, in a cross-sectional study conducted in Uttar Pradesh, researchers observed that 25.5% of the women who were high-risk were illiterate [12]. Our findings could be attributed to the semi-urban distribution of the study population. 

We found that increased antenatal risk scores correlated with poor maternal outcomes. The study showed an increase in the risk of miscarriages in women with moderate- and high-risk pregnancies (10, 52.6%) (6, 31.6%) respectively, and these findings were in line with the results from another study [12]. A retrospective cohort study in Saudi Arabia noted an increase in maternal bleeding in HRPs [23]. Other studies by Prajapati et al. and Kumar et al. found that antepartum bleeding was highest in the high-risk group compared to the low-risk group [12,24]. In this study, we observed similar findings, 5(55.6%) from the high-risk group developed antepartum haemorrhage, considerably higher than the low-risk group 1(11.1%). A study in South India by Pillai et al. found a statistical correlation between HRPs and operative outcomes (65.9% in high-risk vs 25.36% in low-risk pregnancies) [25]. However, our study did not show a difference between the groups (28.9% in high-risk vs 31.8% in low-risk mothers). The risk of developing postpartum haemorrhage was six-fold higher in the high-risk group when compared to the low-risk group, however, this was not statistically significant (66.7%% vs 11.1%). There was a prospective cohort study that reported similar findings and found a statistically significant difference in postpartum bleeding in the high-risk group [25]. 

Adverse neonatal outcomes were correlated according to the risk groups. HRPs were associated with a higher incidence of IUDs and this association was statistically significant (p<0.05). A prospective study conducted in South India revealed a similar higher incidence of perinatal mortality in HRPs [15]. In our study, the relationship between HRPs 13(52.0%) and LBW babies was significant (p<0.05). Other studies have also observed an association between LBW babies and HRPs [15,22]. Bansal et al. observed an association between HRPs and adverse neonatal outcomes such as neonatal respiratory distress (8%) and prematurity (12%) [15]. Another study conducted by Pillai et al. found that 27.3% of the babies born prematurely belonged to the high-risk group [25]. Our study found a statistically significant correlation between respiratory distress and higher-risk pregnancies, 10(45.5%) of the neonates who developed respiratory distress belonged to the high-risk group and 9(40.9%) from the moderate-risk group when compared to the low-risk group 3(13.6%) (p<0.05). Out of the 5 neonates who were born prematurely (<37 weeks of gestation), 4(80.0%) were borne by high- and moderate-risk mothers and 1(20%) by a low-risk mother. This was not statistically significant. Regarding the need for NICU admissions, there were four cases of neonates requiring NICU admission, 2 in the high-risk group, 1 in the moderate-risk and 1 in the low-risk group, showing no statistical significance. However, studies conducted in Karnataka and Kerala reported adverse fetal outcomes with the need for NICU admissions being more prevalent in HRPs (29.5% and 27.3%), respectively [24,25].

Limitations 

Since the study was conducted at the level of a single secondary health centre, the findings of the study are not strictly representative of the population in a larger setting. 

## Conclusions

A significant number of pregnant women are at risk of developing complications leading to adverse maternal and neonatal outcomes. The findings of this study conclude that 18.3% of the total pregnancies belong to the high-risk category when stratified using modified Cooplands scoring tool and would benefit from targeted obstetric care. Nulliparity is a contributory risk factor to HRPs, and these pregnancies were significantly associated with adverse outcomes such as miscarriages, bleeding complications and increased need for operative delivery in mothers. Adverse neonatal outcomes associated were LBW status and respiratory distress.
